# Induction of lymphangiogenesis in and around axillary lymph node metastases of patients with breast cancer

**DOI:** 10.1038/sj.bjc.6603443

**Published:** 2006-10-31

**Authors:** G G Van den Eynden, I Van der Auwera, S J Van Laere, V Huygelen, C G Colpaert, P van Dam, L Y Dirix, P B Vermeulen, E A Van Marck

**Affiliations:** 1 Translational Cancer Research Group Antwerp(Lab Pathology University Antwerp/University Hospital Antwerp, Edegem; Oncology Center, General Hospital St-Augustinus, Wilrijk), Antwerp, Belgium

**Keywords:** lymphangiogenesis, lymph node metastases, podoplanin, D2-40, breast cancer

## Abstract

We studied the presence of lymphangiogenesis in lymph node (LN) metastases of breast cancer. Lymph vessels were present in 52 of 61 (85.2%) metastatically involved LNs *vs* 26 of 104 (25.0%) uninvolved LNs (*P*<0.001). Furthermore, median intra- and perinodal lymphatic endothelial cell proliferation fractions were higher in metastatically involved LNs (*P*<0.001). This is the first report demonstrating lymphangiogenesis in LN metastases of cancer in general and breast cancer in particular.

Lymph node (LN) status is the most important prognostic factor for patients with breast cancer. The presence and the extent of axillary LN metastases reflect the probability that the cancerous process has spread through the body and both are strongly correlated with the development of distant metastases and with shortened disease-free and overall survival. Lymph node metastases are more than passive tumour deposits. Metastatic tumour sites are capable of inducing a vascular stroma and can actively contribute to tumour progression and to further metastatic spread. To what extent processes involved in progression of primary tumours, such as angiogenesis and lymphangiogenesis, contribute to progression of secondary sites is largely unknown. Reports have suggested differences between primary tumours and secondary sites and between different secondary sites. Whereas primary breast tumours grow angiogenesis dependently, we demonstrated that 90% of breast cancer liver metastases grow according to an angiogenesis-independent replacement pattern (Stessels *et al*, 2004). The growth of breast cancer LN metastases, on the contrary, was angiogenesis dependent and angiogenesis and hypoxia in the metastases were correlated with angiogenesis and hypoxia in the primary tumours (Van den Eynden *et al*, 2005). Guidi *et al* (2000) demonstrated that the presence of vascular hot spots in LN metastases, but not in the primary breast tumours was associated with decreased survival.

In the present study, we compared the expression of the lymphatic endothelium-specific markers Prox-1, LYVE-1 and podoplanin in metastatically involved and uninvolved LNs of patients with breast cancer. Prox-1 and LYVE-1 are, respectively, a transcription factor and a hyaluronan receptor that show specificity for lymphatic endothelial cells. D2-40 was originally described as a selective monoclonal antibody to a *M*_r_ 40 000 O-linked sialoglycoprotein that reacts with a fixation-resistant epitope in lymphatic endothelium (Kahn and Marks, 2002). Recently, the D2-40 antibody has been shown to specifically recognise podoplanin, a glomerular podocyte membrane protein (Schacht *et al*, 2005) and has been shown to be a very sensitive and specific marker for lymphatic endothelium in most tissues (Evangelou *et al*, 2005) and especially in breast cancer (Van der Auwera *et al*, 2005). We investigated the presence and extent of lymphangiogenesis in LN metastases of breast cancer using the podoplanin antibody.

## MATERIALS AND METHODS

### Patients and samples

One hundred and ten patients with operable breast cancer were included in this study, 49 patients with LN-negative and 61 patients with LN-positive breast cancer. Clinico-pathological features are compared between both study groups in Table 1, using the UICC TNM system. Formalin-fixed paraffin-embedded tissue blocks of one metastatically involved and – if available – one uninvolved LN of patients with LN-positive breast cancer and one metastatically uninvolved LN of patients with LN-negative breast cancer were selected for immunohistochemical examination. Of six LN-positive patients, no uninvolved LN was available.

### Expression of lymphatic endothelium-specific and vascular markers

**Figure 1:**
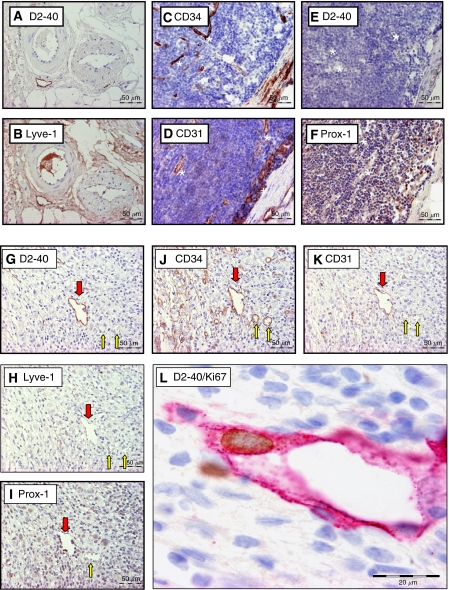
Overview of the immunohistochemical stains used on serial sections of a metastatically uninvolved (perinodal fat: **A**–**B**; LN: **C**–**F**) and involved LN (**G**–**L**). In uninvolved LNs, high endothelial venules (^*^) show CD34 (**C**) and CD31 (**D**) reactivity. Littoral cells (+) are positive for CD31 (**D**) and Prox-1 (**F**). In metastatically involved LNs, podoplanin-positive LVs (red arrow) are demonstrated (**G**). Blood vessels (yellow arrow) are podoplanin negative (**G**). The LVs show CD34 (**J**), CD31 (**K**) and Prox-1 (**I**) reactivity. Although in perinodal fat (**B**) LVs are strongly positive for LYVE-1, the LVs in metastatically involved LNs are only faintly positive for LYVE-1 (**H**). Furthermore, a proliferating lymphatic endothelial cell is shown at the border of a LN metastasis (**L**) (LN: lymph node; LV: lymph vessel).

Immunohistochemical stainings for the lymphatic endothelium-specific markers podoplanin (clone D2-40, Dako, Glöstrup, Denmark), Prox-1 (polyclonal, Reliatech, Braunschweig, Germany) and LYVE-1 (polyclonal, Reliatech) and for the panendothelial markers CD34 (clone QBEnd10, Dako) and CD31 (clone JC70A, Dako) were performed on serial sections of a subset (*n*=20) of metastatically involved and uninvolved LNs. A detailed description of the protocol of these stainings has been published before (Van der Auwera *et al*, 2005) (Figure 1).

### Presence of lymph vessels and lymphangiogenesis

An immunohistochemical staining for podoplanin (clone D2-40, Dako) and an immunohistochemical double staining with anti-podoplanin (clone D2-40, Dako) and anti-Ki-67 antibodies (clone MIB-1, Dako) were performed on sections of all included cases. The presence of lymph vessels (LVs) was assessed intranodally (within the LN capsule) of metastatically involved and uninvolved LNs, and, if LVs were present, intranodal lymphatic endothelial cell proliferation fraction (LECP%) was assessed by counting Ki-67 positive and negative lymphatic endothelial cells. If perinodal LVs were present, perinodal LECP% was also assessed. Only cases with >10 lymphatic endothelial cells were included for statistical analysis.

### Statistical analysis

Statistical analyses were performed with the SPSS 13.0 software package. A *P*-value <0.05 was considered statistically significant. Correlations between categorical variables (e.g. presence of LVs, metastatical involvement) were analysed using a *χ*^2^ test. LECP% in different groups was compared with a Kruskal–Wallis nonparametric test.

## RESULTS

Results of the comparison of the expression of the different vascular markers in and around metastatically uninvolved LNs of patients with breast cancer are shown in Table 2. Although the littoral cells of the marginal and trabecular sinuses were very focally and faintly immunoreactive for podoplanin, no intranodal podoplanin immunoreactive vascular structures were demonstrated except in trabecular septa or areas of lipomatosis and fibrosis (Figure 1A–F). In contrast, in most metastatically involved LNs vascular structures staining for podoplanin were observed. These structures were also positive for Prox-1, CD31 and CD34. They were only faintly LYVE-1 positive or did not show LYVE-1 immunoreactivity (Figure 1G–K).

We then analysed the presence of LVs in the total study population, using podoplanin as a lymphatic marker. Lymph vessels were only seen in 26 out of 104 (25.0%) uninvolved LNs. In 22 of these 26 (84.6%) LNs, LVs were seen in areas of lipomatosis or fibrosis or in trabecular septa. There was no difference in the presence of LVs in uninvolved LNs of patients with LN-negative and LN-positive breast cancer (*P*=0.91). In contrast, LVs were demonstrated in 52 of 61 (85.2%) metastatically involved LNs (*P*<0.001) (Table 3). These LVs were often localised at the metastasis/LN interface or in the LN parenchyma close to the metastasis.

**Figure 2:**
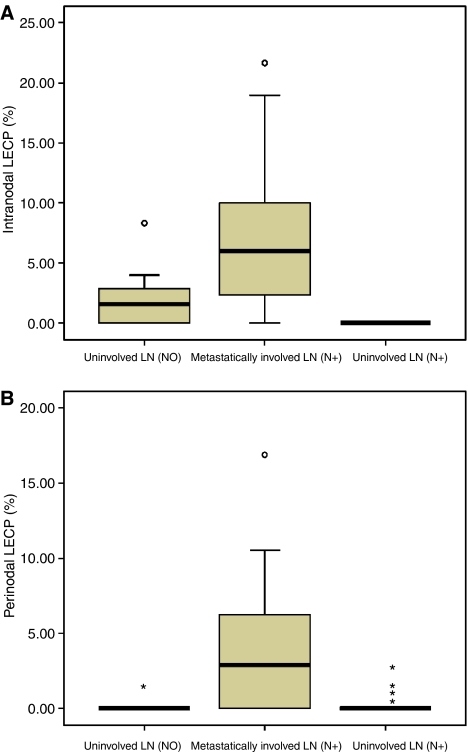
Comparison of intranodal (**A**) and perinodal (**B**) LECP% between metastatically involved and uninvolved axillary LNs in patients with LN-negative and LN-positive breast cancer. Both intra- (*P*<0.001) and perinodal (*P*<0.001) LECP% is significantly increased in metastatically involved LNs compared to uninvolved LNs (LECP%: lymphatic endothelial cell proliferation fraction; LN: lymph node; N0: lymph node negative; N+: lymph node positive).

Figure 2 shows Box and Whisker's plots comparing intranodal (Figure 2A) and perinodal (Figure 2B) LECP% in all groups. The median intranodal LECP% was 6.0% (*n*=42) in metastatically involved LNs compared to 1.6% (*n*=11) in uninvolved LNs of LN-negative and 0.0% in uninvolved LNs (*n*=9) of LN-positive patients (*P*<0.001). The median perinodal LECP%s were 2.9, 0.0 and 0.0% in metastatically involved LNs (*n*=25), uninvolved LNs from LN-positive patients (*n*=37) and in uninvolved LNs from LN-negative patients (*n*=20), respectively (*P*<0.001).

## DISCUSSION

To the best of our knowledge, this is the first report demonstrating the presence of lymphangiogenesis in secondary sites of human cancer in general and in LN metastases of breast cancer in particular. First, different lymphatic endothelial-specific markers were compared. As LYVE-1 reactivity of LVs close to LN metastases decreases, as LYVE-1 reactivity of macrophages and intravascular proteins in both blood and LVs hampers interpretation (see Figure 1) and as Prox-1 is a nuclear antigen, we preferred to use podoplanin (clone D2-40) to study the presence of LVs and lymphangiogenesis in the total study population. In a previous study, we have shown that although LYVE-1 expression on LVs located at the tumour periphery was strong, no or weak immunoreactivity was found in intratumoral LVs and that podoplanin is the best marker to visualise LVs in and around primary breast tumours (Van der Auwera *et al*, 2005).

In primary human tumours, the contribution of lymphangiogenesis *vs* lymphatic cooption and the functionality of tumour lymphatics are still controversial. Nevertheless, LVs containing proliferating nuclei have been observed in breast cancer (Van der Auwera *et al*, 2005), endometrial cancer (Koukourakis *et al*, 2005), head and neck cancer (Beasley *et al*, 2002) and melanoma (Dadras *et al*, 2003; Straume *et al*, 2003). As LVs were only found in a minority of uninvolved axillary LNs, the increase of the presence of LVs in metastatically involved LNs is likely to be due to lymphangiogenesis. An increased LECP% was indeed found in and around metastatically involved LNs. Although misinterpretation of nuclei from proliferating tumour cells penetrating the vessel wall or underlying the LV as endothelial cell nuclei might lead to falsely elevated LECP%, careful interpretation by experienced investigators can largely reduce this bias. Tumour cell nuclei and endothelial cell nuclei mostly differ from shape and the use of microscopy at different focal planes further reduces this bias. The methodology adopted in this study to measure ECP% is widely accepted in the angiogenesis field (Vermeulen *et al*, 2002) and has recently been introduced in the lymphangiogenesis field (Dadras *et al*, 2003; Beasley *et al*, 2002; Straume *et al*, 2003; Koukourakis *et al*, 2005; Van der Auwera *et al*, 2005). The median intranodal LECP% of 6.0% suggests that lymphangiogenesis in metastatically involved LNs is higher than in primary breast tumours: we previously demonstrated that LECP% in primary breast tumours was 1.83% (Van der Auwera *et al*, 2005).

Our results corroborate the concept of LN lymphangiogenesis, which was recently introduced and shown to be involved in the recruitment of dendritic cells to inflamed LNs (Angeli *et al*, 2006). The authors demonstrated that the lymphangiogenic response in the LNs was particularly localised in the subcapsular space and around B-cell follicles from where LVs penetrated into the cortex. Whether the outgrowth of new LVs in metastatically involved axillary LNs originates from the subcapsular marginal sinus or from LVs in areas of lipomatosis or in fibrous septa remains to be elucidated.

Different hypotheses could be raised about the role of LN lymphangiogenesis in metastatically involved LNs. On the one hand, LN lymphangiogenesis might be part of the immunological reaction against the tumour cells and on the other hand be involved in tumour progression and metastases, as in primary tumours. Therefore, LN lymphangiogenesis, as LN angiogenesis, might contribute to the metastatic spread of breast cancer. In a transgenic mouse model of skin cancer, Hirakawa *et al* (2005) demonstrated that the induction of lymphangiogenesis by vascular endothelial growth factor A is involved in tumour progression. They furthermore showed that VEGF-A also induces lymphangiogenesis in the sentinel LN and that lymphangiogenesis is induced before metastasising (Hirakawa *et al*, 2005). Based on our data, it is difficult to investigate the mutual contribution of local and remote mechanisms to the induction of lymphangiogenesis in LNs of patient with breast cancer. However, the difference in LECP% in uninvolved LNs of patients with N0 *vs* N+ breast cancer is intriguing in this context.

## Figures and Tables

**Table 1:** Comparison of clinico-pathological variables of the primary tumour in N0 and N+ patients included

	**N0 (*n*=49)**	**N+ (*n*=61)**	***P*-value**
Mean age (range)	60.9 (34.3–85.4)	59.6 (25.3–84.4)	0.63
			
*Histology*			0.81
IDA	40	48	
ILA	6	10	
Other	3	3	
			
*T status*			0.004
T1	28	15	
T2	18	37	
T3	2	5	
T4	1	4	
			
*N status*			NA
N0	49	0	
N1	0	27	
N2	0	20	
N3	0	14	
			
*ER*			0.78
Negative	14	16	
Positive	35	45	
			
*PR*			0.26
Negative	18	29	
Positive	31	32	
			
*Her-2/neu*			0.6
Negative	42	50	
Positive	7	11	

ER=oestrogen receptor; IDA=infiltrating ductal adenocarcinoma; ILA=infiltrating lobular adenocarcinoma; N0=lymph node negative, N+=lymph node positive; PR=progesteron receptor.

Age, histological type, T and N status, ER and PR status, HER-2/neu oncoprotein status were recorded by review of the pathology files. In N+ patients, tumours were larger than in N0 patients.

**Table 2:** Expression of different vascular markers in metastatically uninvolved LNs of patients with breast cancer

	**CD31**	**CD34**	**Podoplanin**	**Prox-1**	**LYVE-1^a^**
High endothelial venules	+	+	−	−	−
Littoral cells (lining subcapsular and trabecular sinuses)	+	−	Very focally and faintly	+	Faintly
					
*Fibrous capsule and trabecular fibrous septa*					
BV	+^b^	+^b^	−	−	−
LV	+^b^	+^b^	+	+	+
					
*Fibrous and lipomatous areas*					
BV	+^b^	+^b^	−	−	−
LV	+^b^	+^b^	+	+	+
					
*Perinodal fat*					
BV	+^b^	+^b^	−	−	−
LV	+^b^	+^b^	+	+	+

BV=blood vessel; LV=lymph vessel.

a LYVE-1 positivity of macrophages and of intravascular proteins often hampers interpretation.

b CD31 and CD34 expression of lymphatic endothelial cells is mostly fainter than expression of blood vessel endothelial cells.

**Table 3:** Comparison of the presence of LVs between metastatically involved and uninvolved LNs

	**Presence of LVs**	
	**No**	**Yes**
*Uninvolved LN*		
N0 breast cancer	37	12
N+ breast cancer	41	14
Metastatically involved LN	9	52

LN=lymph node; LV=lymph vessel, N0=LN negative; N+=LN positive.

Uninvolved LNs are divided in LNs from N0 and N+ patients with breast cancer.

*P*-value (metastatically involved *vs* uninvolved LNs) <0.001.

*P*-value (uninvolved LNs N0 *vs* N+)=0.91.
